# Transitions in information processing dynamics at the whole-brain network level are driven by alterations in neural gain

**DOI:** 10.1371/journal.pcbi.1006957

**Published:** 2019-10-15

**Authors:** Mike Li, Yinuo Han, Matthew J. Aburn, Michael Breakspear, Russell A. Poldrack, James M. Shine, Joseph T. Lizier

**Affiliations:** 1 Centre for Complex Systems, The University of Sydney, Sydney, Australia; 2 Brain and Mind Centre, The University of Sydney, Sydney, Australia; 3 Complex Systems Research Group, Faculty of Engineering, The University of Sydney, Sydney, Australia; 4 QIMR Berghofer Medical Research Institute, Queensland, Australia; 5 Department of Psychology, Stanford University, Stanford, California, United States of America; Ernst-Strungmann-Institut, GERMANY

## Abstract

A key component of the flexibility and complexity of the brain is its ability to dynamically adapt its functional network structure between integrated and segregated brain states depending on the demands of different cognitive tasks. Integrated states are prevalent when performing tasks of high complexity, such as maintaining items in working memory, consistent with models of a global workspace architecture. Recent work has suggested that the balance between integration and segregation is under the control of ascending neuromodulatory systems, such as the noradrenergic system, via changes in neural gain (in terms of the amplification and non-linearity in stimulus-response transfer function of brain regions). In a previous large-scale nonlinear oscillator model of neuronal network dynamics, we showed that manipulating neural gain parameters led to a ‘critical’ transition in phase synchrony that was associated with a shift from segregated to integrated topology, thus confirming our original prediction. In this study, we advance these results by demonstrating that the gain-mediated phase transition is characterized by a shift in the underlying dynamics of neural information processing. Specifically, the dynamics of the subcritical (segregated) regime are dominated by information storage, whereas the supercritical (integrated) regime is associated with increased information transfer (measured via transfer entropy). Operating near to the critical regime with respect to modulating neural gain parameters would thus appear to provide computational advantages, offering flexibility in the information processing that can be performed with only subtle changes in gain control. Our results thus link studies of whole-brain network topology and the ascending arousal system with information processing dynamics, and suggest that the constraints imposed by the ascending arousal system constrain low-dimensional modes of information processing within the brain.

## Author summary

Higher brain function relies on a dynamic balance between functional integration and segregation. Previous work has shown that this balance is mediated in part by alterations in neural gain, which are thought to relate to projections from ascending neuromodulatory nuclei, such as the locus coeruleus. Here, we extend this work by demonstrating that the modulation of neural gain parameters alters the information processing dynamics of the brain regions of a biophysical neural model. Specifically, we find that subcritical dynamics in the phase space of neural gain parameters are characterized by high Active Information Storage, whereas supercritical dynamics in this phase space are associated with an increase in inter-regional Transfer Entropy. Our results suggest that the modulation of neural gain via the ascending arousal system may fundamentally alter the information processing mode of the brain, which in turn has important implications for understanding the biophysical basis of cognition.

## Introduction

Although there is a long history relating individual brain regions to specific and specialized functions, regions in isolation cannot perform meaningful physiological or cognitive processes [1]. Instead, starting at a lower scale, a few prominent features of the brain’s basic mechanisms stand out. Firstly, neurons exist in vast numbers, each acting as an individual element with a similar set of rules. Secondly, the response of individual neurons to stimuli are far from linear—small changes in the surrounding milieu can lead to abrupt changes in neural dynamics [2]. Thirdly, all neurons interact with other neurons through synapses, and hence form a network that spans the central nervous system [3]. Furthermore, this structural backbone supports coherence of physiological activity at larger scales, giving rise to distributed functional networks [4]. Therefore, in every regard, the brain is a complex system whose computational power stems from the emergent properties of coordinated interactions between its components [5, 6]. Understanding how the topology *and* dynamics of these networks give rise to its function is one of the most central questions that computational neuroscience aims to address.

From comparing a range of physical and mathematical systems, it is known that complex systems can exist in multiple distinct phases. For instance, groups of water molecules can exist as a solid, liquid or gas, depending on the surrounding temperature and pressure. By altering one or more tuning parameters (e.g. temperature in the water example), the system can cross clearly defined critical boundaries in the parameter space. These critical transitions are typically abrupt and often associated with qualitative shifts in the function of a system (e.g. consider the stark differences between ice and liquid water). They are often of great interest due to their ubiquity and the implications for systemic flexibility [8].

**Fig 1:**
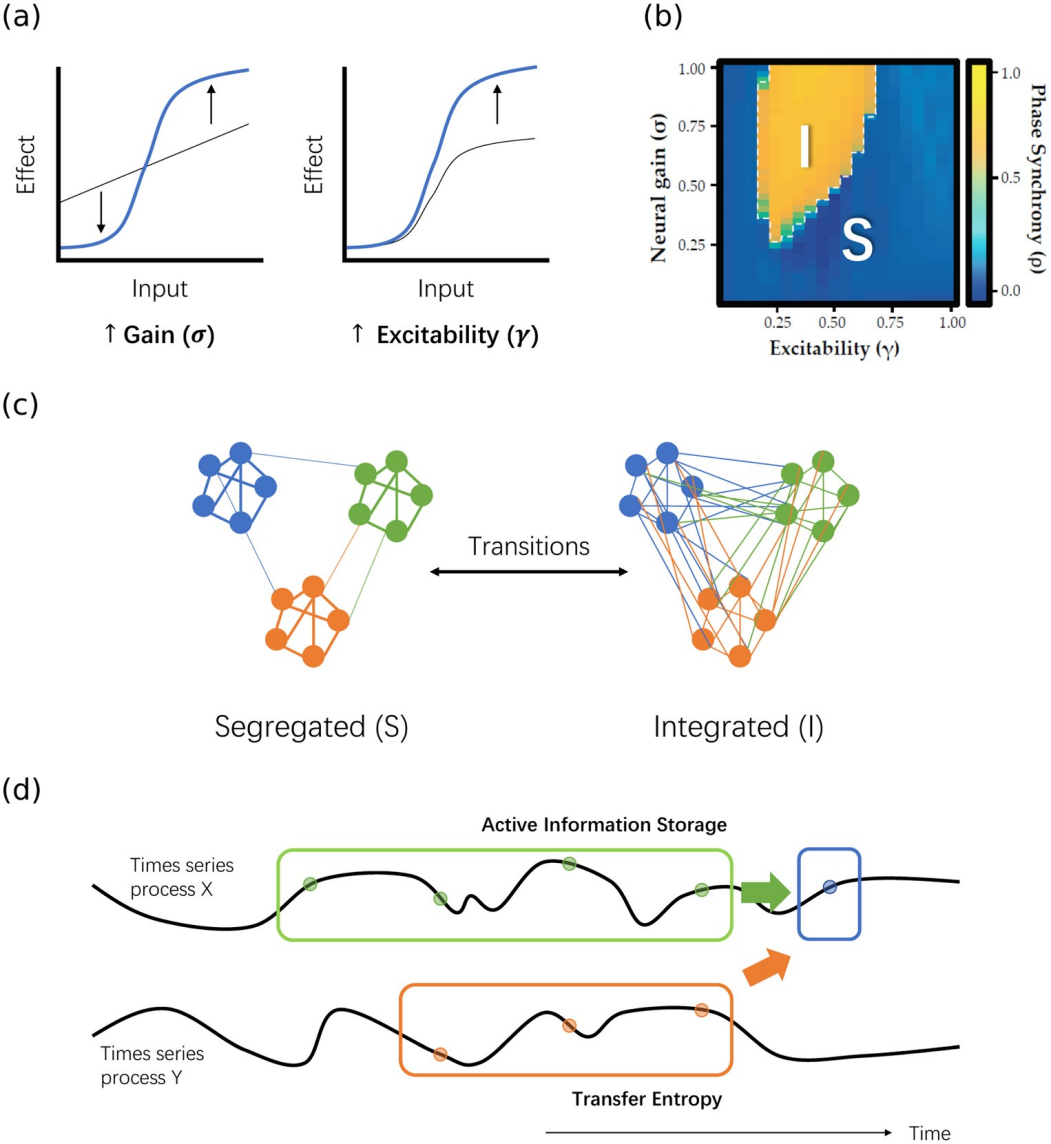
Schematic diagram showing how neural gain parameters (e.g. under modulation by noradrenaline) may potentially affect the information processing structure of the brain. (a) The effect of neural gain (*σ*) and excitability (*γ*), the two tuning parameters being varied in our neural mass model (see Methods), on the response of individual neurons to stimuli are shown schematically. Each input stimulus to a target region in the model contributes an effect to the rate of change of the target via a sigmoid function. Arrows in the figures indicate how the sigmoid function defining these effects changes with increases in these gain parameters (with *σ* increasing nonlinearity of response and *γ* increase amplification). (b) Previous results from [7] (adapted under Creative Commons Attribution License CC BY 4.0) showing that varying neural gain and excitability may cause abrupt changes in the mean phase synchrony of the brain from modelled fMRI BOLD recordings, implying the existence of a critical boundary between a segregated phase (“S”, low phase synchrony) and an integrated phase (“I”, high phase synchrony) in the brain. (c) Schematic diagram of functional brain networks in the segregated and integrated phases, and how changing neural gain and excitability may lead to transitions between the two. (d) Schematic diagram of the concept of active information storage and transfer entropy, and how they may be affected by phase transitions. Qualitatively, active information storage (green arrow) describes information on the next instance *X*_*n*+1_ (blue sample) of a time series *X* provided by its own history ($X_{n}^{\left(k\right)}$, green samples), whereas transfer entropy (orange arrow) describes that provided by the past ($Y_{n}^{\left(l\right)}$, orange samples) of another time series *Y* in the context of the target’s history. See further details on these measures in Methods.

Empirical observations in neural cultures, EEG and fMRI recordings provide evidence that the brain operates near criticality [8–16]—one form of which is a transition between two distinct states in the functional network topology [17] (see Fig 1c). At one extreme, different regions of the brain are highly segregated, and each region prioritizes communication within its local topological neighbourhood. At the other pole, the whole brain becomes highly integrated, and cross-regional communication becomes far more prominent. Experimentally, the resting brain is found to trace a trajectory between the two states, and can transition abruptly into the highly integrated state when the subject is presented with a cognitively challenging task [18].

As described in Methods regarding [7], this transition across a critical boundary can be achieved in a neural mass model by tuning two neural gain parameters: the neural gain *σ* and excitability *γ*. (From this point onwards, if “neural gain” refers only to the *σ* parameter rather than the two parameters collectively, then this is indicated by including *σ* in such text). Fig 1a shows how the gain parameter *σ* increases each region’s signal-to-noise ratio by altering the shape (or non-linearity) of the input-output curve, while the excitability *γ* scales the magnitude or amplification of the response. Biologically, the control parameter could plausibly be implemented through subtle alterations in the concentration of ionotropic and metabotropic neuromodulatory neurotransmitters at the level of neural circuits [19, 20]. Of these neurochemicals, dynamic changes in noradrenaline, mediated by ascending noradrenergic projections from the pontine locus coeruleus, have been shown to play a particularly important role in the modulation of the precision and responsivity (i.e., the ‘neural gain’) of targeted neurons [7, 21, 22]. The result of tuning neural gain parameters in the neural mass model can be characterized via temporal measures of the activity in each region, such as the average phase synchrony order parameter. As shown in Fig 1b, the model displays two distinct dynamic states in modelled fMRI BOLD recordings, one exhibiting high phase synchrony between the dynamics of all brain regions and the other exhibiting low phase synchrony in these dynamics. Fig 1b also shows that the sharp transition in mean synchrony requires only a small change in gain parameters, which is an illustration of the critical behaviour of the network in that region of parameter space. From another perspective, the result of tuning neural gain parameters can be seen in significant alterations to the functional network topology of the brain, as characterized by graph theoretical parameters such as the mean participation coefficient. These functional network measures provide the interpretation of integrated (high phase synchrony) versus segregated (low phase synchrony) states alluded to above (Fig 1c).

A question then arises: why might it be favourable for the brain to be in a near or quasi-critical state between these regimes in the first place? Specifically, are there computational advantages accompanying this structure? Many have proposed that this signature may reflect an evolutionary optimisation, allowing for both an effective balance between long- and short-range interactions between neural regions, as well as rapid transitions between segregated and integrated states [23, 24]. For instance, a highly segregated brain cannot communicate effectively to share information across different sub-networks, while a highly integrated state results in homogeneity in the flow of signals and a reduction in meaningful interactions, as in the case of epilepsy [18]. Hence, an optimal state for the brain is likely a flexible balance between the two extremes. Indeed, systems poised near criticality are well-known to exhibit other distinct characteristics which could be usefully exploited in the brain, such as increased autocorrelation times and variance [25–27], increased coupling across the system [28, 29] and maximal sensitivity to tuning parameters [30].

Prompted by early conjecture [31], there is evidence from neural recordings and studies of other complex systems that phase transitions are often related to changes in the *information processing* structure of a system. Shew et al. [10] demonstrated maximal information capacity (via entropy) and sharing of information (via mutual information) near critical transitions in dynamics of neural cultures, with the transitions investigated by manipulating excitation-inhibition ratios. Although the study referred to the latter measure as “information transmission”, the mutual information remains a measure of statically shared information, and information transmission and processing in general are more appropriately modelled with measures of dynamic state updates [32]. These measures of “information dynamics” model the interacting entities in the system as computational units, translating the intrinsic dynamics of their state updates into statistical models of how information is stored within or transferred between these entities as they update their state in time [32, 33]. Such measures have provided more direct evidence of changes in information processing structure associated with phase transitions in the brain, in preliminary results of Priesemann et al. [34], and in other complex systems. In artificial recurrent neural networks for example, both information transfer and storage were observed to be maximized close to a critical phase transition (with respect to perturbation propagation in reservoir dynamics) [35], suggesting that these intrinsic information processing advantages underpinned the known [36] higher performance of similar networks near the critical point on various computational tasks. These changes in information processing structure can also explain some of the aforementioned characteristics near the critical point, such as increased autocorrelation times (as a result of elevated information storage) and coupling (as a result of increased information transfer). Similar results are seen in the well-known phase transition with respect to temperature in the Ising model, with information transfer maximized near the critical regime [37]. Furthermore, the dynamics of Boolean networks (models for gene regulatory networks [38]) exhibiting order-chaos phase transitions are dominated by information storage in the ordered low-activity phase, and information transfer in the high-activity chaotic phase [39, 40]. At the critical regime, networks exhibit a balance by combining relatively strong capabilities of both information storage and transfer. This transition in Boolean networks can be triggered either by directly altering the level of activity in the dynamical rules of the nodes, or by sweeping the randomness in network structure starting with a regular lattice network (low-activity) through small-world [41] and onto random structure (high-activity). The dynamics of the brain, being a highly analogous system to these exhibiting phase transitions between functional segregation and integration, may exhibit similar patterns in information storage and transfer capabilities near the critical regime, and on both sides of the critical boundary. Hence, we aim to examine the quantitative changes in the information processing properties of the brain under the framework of information theory.

From a Shannon information-theoretic perspective, we measure information as the reduction in uncertainty about an event with an unknown outcome [42]. For a given time series process within a larger system, such as the blood oxygen level dependent (BOLD) data for a single voxel in the brain (i.e. the smallest identifiable region in an fMRI scan), the sources of information regarding the next event in the process include the history of the time series of the process itself, and the history of other processes in the system as inputs, such as the time series of other voxels. Here, within the framework of information dynamics [32] we model the amount of information storage as that provided from within a time series process using the active information storage (AIS) [43]. We model information transfer as that provided by another source to a target process, in the context of the target past, using transfer entropy (TE) [44]. Fig 1d provides a simple illustration of this concept.

By using computational modelling to examine the behaviour of these two information-theoretic measures across a parameter space of varying values of neural gain and excitability, we aim to address two main questions: firstly, are there differences in the information processing structure as a function of alteration of neural gain parameters? And secondly, does a quasi-critical state provide computational information processing benefits? Given the properties of the previously determined topological measures (Fig 1b) and how they relate to previous results on information processing around critical regimes, we predicted differential information processing structures across the parameter space of gain and excitability, and hypothesized that: i. the active information storage across the system should be maximized in the subcritical region before the critical boundary, whereas ii. the transfer entropy would be maximized after the boundary in the supercritical region, and iii. that storage and transfer should be relatively balanced at the critical transition. A change in information processing near criticality may allow for rapid alterations in the balance between states dominated by information storage in the subcritical phase and information transfer in the supercritical phase, hence providing flexibility for the dynamical structure of the brain to quickly adapt to and complete a wide range of tasks.

## Results

Regional time series of neuronal dynamics were generated by a 2-dimensional neural oscillator model with stochastic noise [45] built on top of a weighted, directed white matter connectome [46], simulated with the Virtual Brain toolbox [47]. The properties of inter-regional coupling were systematically adjusted using the parameters for gain (*σ*) and excitability (*γ*) (see Methods for more details).

In contrast to the previous study which used the same underlying generative model [7], we did not transform the raw data into a simulated BOLD signal. Instead, information-theoretic measures were calculated directly on each region’s average membrane voltage *V* (which is monotonically related to the neural firing rate, see Methods)—sampled at a rate of 2 kHz. This allowed us to construct a model of information processing that was more closely linked with the underlying dynamics of the neural system. For each point in the two dimensional *σ*-*γ* parameter space, information storage was calculated for each region from its own generated time series process. Similarly, information transfer was calculated for each directed pair of regions from their own generated time series processes at each point in the *σ*-*γ* space. The fast sampling rate obliged us to apply the information-theoretic measures treating the data as arising from a continuous-time process (see Methods).

### Information storage peaks in the subcritical region at intermediate *γ*

The active information storage of a process measures the extent to which one can model the next sample of a time series as being computed from (a time-delay phase space embedding of) its past history [43] (see Methods). High active information storage implies that the past states of a process are strongly predictive of the next observation. For this experiment, we measured the active memory utilization rate (AM rate), which is a formulation of active information storage suitable for continuous time processes [48] (see Methods).

**Fig 2:**
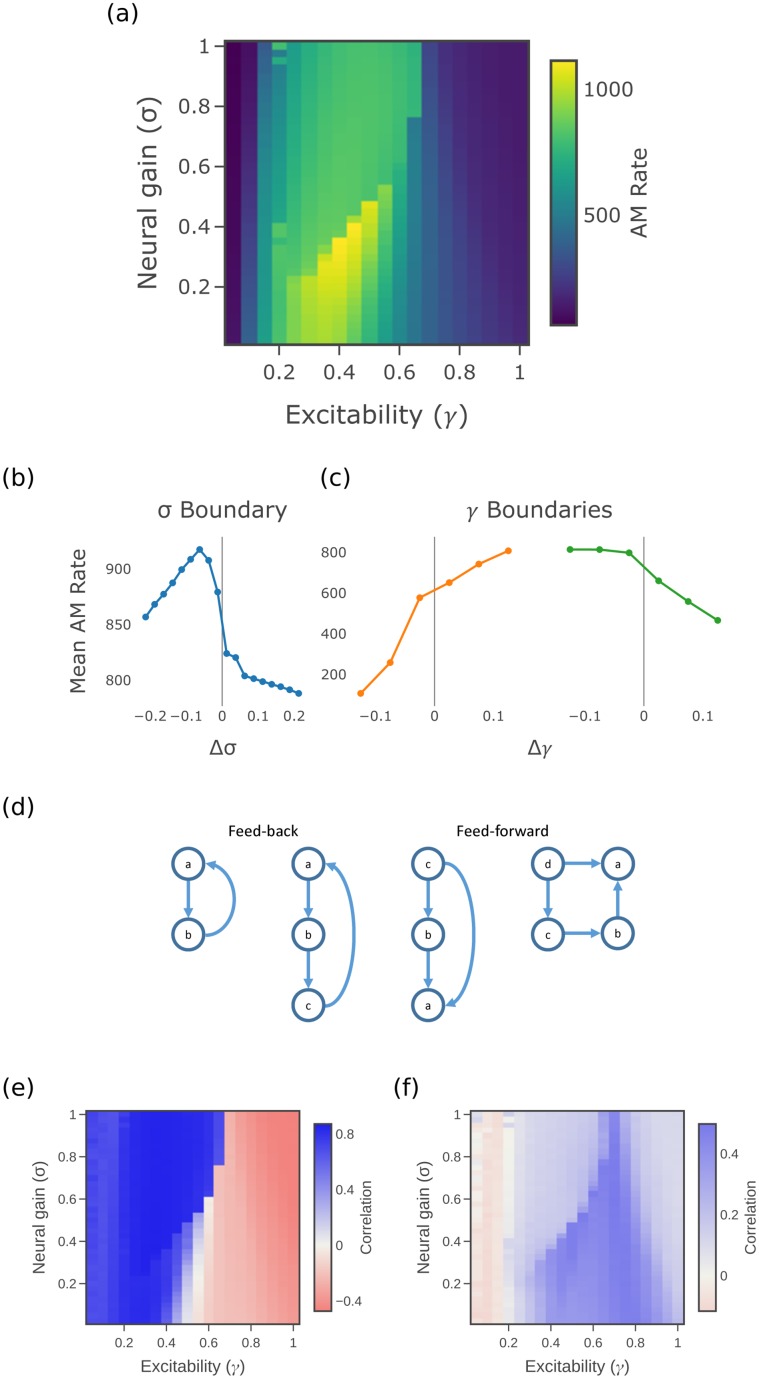
Measures of information storage. (a) Active memory utilization rate. (b) and (c) Mean active memory rate across *σ* and *γ* phase boundaries. (d) Network motifs supporting information storage in the dynamics of node *a*. (e) Correlation of AM rate to local network support (weighted motif counts). (f) Correlation of AM rate to normalized within-region synaptic connection weight. Matching colour scale is used for (e) and (f). By convention we use blue-white-red color scale for correlation plots to emphasise the positive-negative distinction, and default blue-yellow scale for other plots.

Fig 2a plots the active memory rate (averaged over all regions) with respect to the *σ*-*γ* space. The time-delay embedding parameters for the past history were set to an embedding dimension of *k* = 25 with embedding delay of *τ* = 12 (see details in Methods). Active memory rate peaks at what was previously identified as the subcritical or segregated regime [7] (compare to regime identification in Fig 1b). We also see two types of transitions that divide the space up into four qualitative regions. One transition occurs over variations in *γ*: the highest information storage occurs at intermediate values of *γ*, with a sharp dropoff on either side. Within this band of intermediate *γ* there is also a transition in *σ*, with the highest information storage occurring at small values of *σ*, again with a sharp dropoff across the critical transition.

This correspondence with the previously identified regions can be observed more clearly from Fig 2b and 2c, which plots the average values across the *σ* and *γ* boundaries, respectively, based on the synchronization order parameter of the model from [7]. A qualitative change is observed at these boundaries, where the active memory rate is highest (and peaking) in the subcritical regime with respect to *σ*, whilst still exhibiting sharp transitions to higher values in the supercritical regime with respect to *γ*.

### Correlation of information storage to motif counts suggests distributed memory in supercritical regime

Information storage in time-series dynamics of network-embedded processes is known to be supported both by mechanisms internal to a node (i.e. self-loops) as well as by distributed network effects [49, 50]. The distributed network effects supporting information storage include recurrent or loop motif structures within directed structural networks, such as low order feed-forward and feedback loops, and under simple coupled Gaussian dynamics an exact relationship can be derived [51]. For the dynamics used with this model, we do not have an analytic derivation of the exact relationship, but use a heuristic to approximate the relative “local network support” provided for information to be stored in the time-series process at a particular region. The local network support for a given region is computed, taking inspiration from [51], as a linear weighted path sum of specific types of motifs (of length larger than 1) involving that region. Full details are provided in Methods; example motifs supporting information storage (i.e. the low-order terms used in the local network support heuristic are shown in Fig 2d. We compute a (Pearson) correlation of this local network support for each region with its active memory rate, at each point in the parameter space, in order to infer where in the parameter space the distributed network effects are a strong factor in the variation of information storage across the regions. As a contrast we also compute a correlation of AM rate to normalized within-region synaptic connection weight (self loop weights *A*_*ii*_ in (3) in Methods), in order to infer where in the parameter space internal (non-network) effects *within* each region are a strong factor in the variation of information storage (across the regions).

Fig 2e shows a high correlation between the active memory rate and local network support in the supercritical phase. This fits with established results of [51] that assume a noise-driven system. This can be contrasted with the correlation of AM rate to normalized within-region synaptic connection weight in Fig 2f, which peaks in the high *γ* subcritical regime. This suggests that primarily synaptic connections internal to a region are the strongest information storage mechanism in the segregated dynamics of the subcritical regime, whilst during the supercritical regime we observe the engagement of network effects via longer motifs to support memory largely via more distributed mechanisms in the integrated dynamics. Interestingly, we note that the subcritical regime with strongest information storage (intermediate *γ*, low *σ*) appears to have support from both within-region synaptic connections and longer motifs.

### Information transfer is maximized in the supercritical region

Information transfer from one process to another is modelled by the transfer entropy [44] as the amount of information which a source provides about a target’s next state in the context of the target’s past (see Methods).

For this experiment on continuous-time processes, we measure the transfer entropy rate [52]. Unless otherwise stated, we constrain the information sources considering only those which are causal information contributors to the target over their source-target time delay (following [39, 53, 54]); these are known from the directed structural connectome and delays used in the simulation (see Methods).

**Fig 3:**
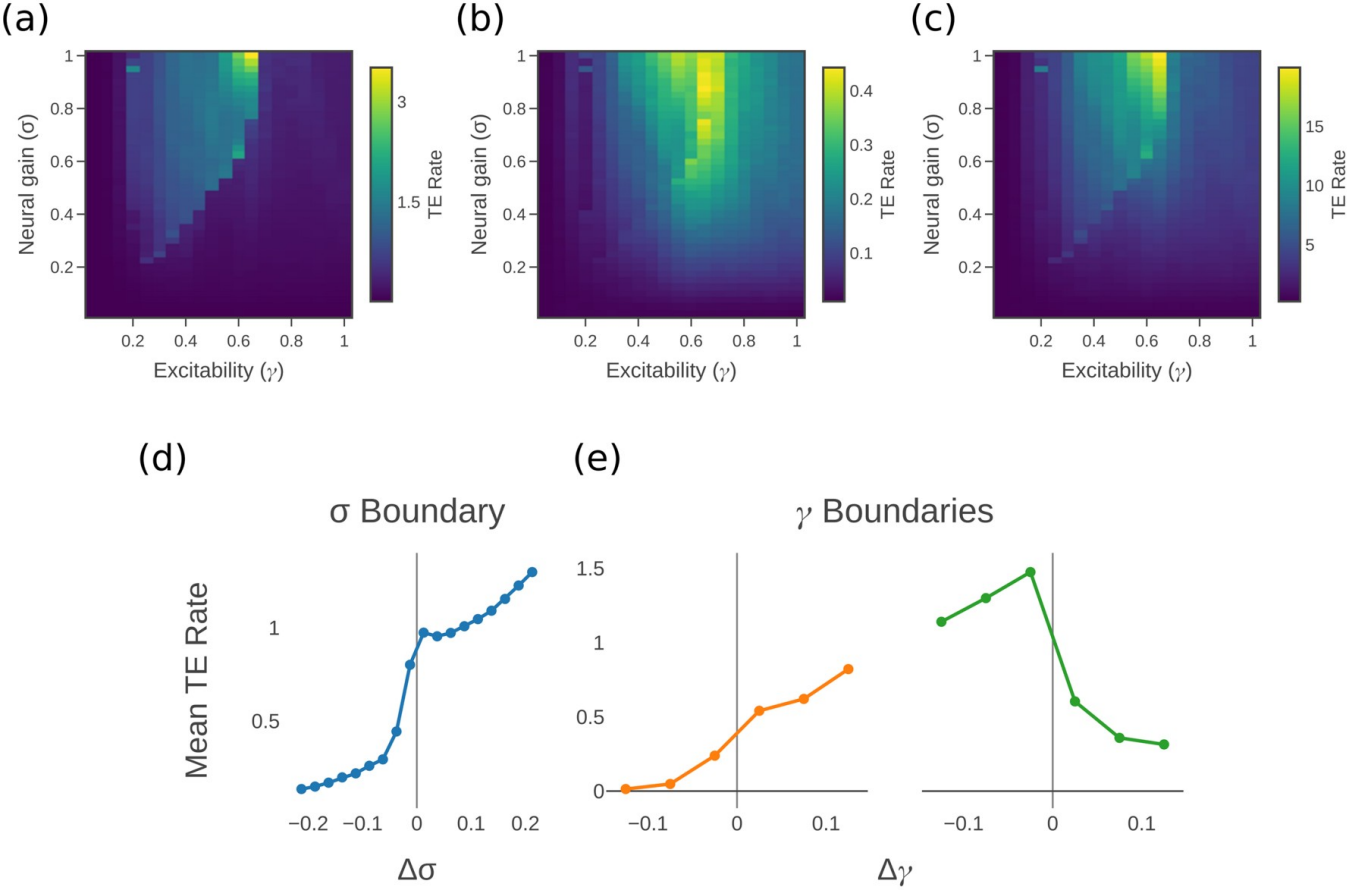
Measures of information transfer. (a) Average transfer entropy rate over causal edges (those connected source → target by the directed connectome). (b) Conditional transfer entropy rate over causal edges. (c) Collective transfer entropy rate of causal edges. (d) and (e) Mean TE rate across *σ* boundary and *γ* boundaries.

For each point in the parameter space of *σ* and *γ*, we calculate all the pairwise transfer entropy rates to targets from each of their causal parents. (There are an average of 19.7 causal parents per source, with a standard deviation of 7.65). These values are averaged across all such directed pairs to give the mean (pairwise) transfer entropy rate across the network at each *σ* and *γ* pair. Fig 3a shows a clear separation of the parameter space into the subcritical and supercritical regions (as defined in the previous work [7], see Fig 3d and 3e), with information transfer occurring almost exclusively in the supercritical or integrated regime. An additional trend within the supercritical region can be seen, with TE rate rising as *σ* and *γ* increases.

Higher order terms for information transfer can also be calculated in addition to the pairwise components. Conditional transfer entropy [55, 56] (see Methods) adds the history of a third process or a collection of processes to be conditioned on in addition to the history of the target itself. For this experiment, we calculate the conditional transfer entropy of the causal source-target relationships, conditioned on all the other causal parents of the target (from the directed structural connectome), and average this across all directed causal pairs. In comparison to the pairwise TE, by conditioning on all the other causal parents the conditional TE captures only the unique information component which this source is able to provide that the others do not, and adds in synergistic or multivariate information about the target that it provides only in conjunction with the set of other sources. Furthermore, any information it holds about the target which is redundant with the other sources is removed.

The collective transfer entropy [56] (see Methods) captures the total transfer of information from a group of sources to a target. We examine the total information from the full set of causal parents to a particular target, and average this across all target processes. The collective TE captures all of the information provided by the sources about the target, whether that information is provided uniquely by any single source, or redundantly or synergistically by some set of them. Importantly, it does not “double count” information held redundantly across multiple sources.

Overall, the mean conditional TE rate in Fig 3b and the mean collective TE rate in Fig 3c show qualitatively similar trends to the pairwise TE rate in Fig 3a, with the same strong mean transfer in the supercritical region and trend towards high *γ* and *σ* within that region. From comparing the peak values of the different figures, it can be seen that substantial redundancies exist in the information held about the target between the different sources. That is, the conditional transfer entropy rate is an order of magnitude lower than the simple pairwise measure—indeed, at these low levels, the conditional TE is far less distinguished in the supercritical region from that in the subcritical region. The comparison to pairwise TE suggests that each causal parent is not able to provide much additional information beyond that already apparent from the other parents. The collective transfer entropy rate, on the other hand is an order of magnitude higher than the simple pairwise measure. This may, however, simply be due to the effect of adding up the transfer entropy rate from the different sources, and the collective transfer entropy divided by the number of sources is not actually higher than the pairwise measure. It is difficult to conclude from this whether there are network level effects that give rise to synergies beyond looking at each region in pairwise fashion (see Methods).

The behaviour of information transfer (Fig 3) was observed to be complementary to the patterns of information storage (Fig 2). This paints a picture of a distinct mode in the dynamics of information processing that switches abruptly as the system moves between the supercritical and subcritical phases. The subcritical or segregated phase effectively contains only information storage dynamics, whereas the supercritical or integrated phase contains a significant level of information transfer. (Subtleties within these phases are described in the Discussion). However, it should be noted that the values for transfer entropy rate are still smaller than the active memory rate by one or two orders of magnitude, even in the collective case. This is partially due to the relative simplicity of our neural mass model, and particularly to the regularity of the oscillations which they produce. The relative changes in storage and transfer separately across the phases though are far more important in understanding the dynamics than the difference in scale when the two are compared.

### Correlation of information transfer with in-degree shows change in behaviour at the phase boundaries

**Fig 4:**
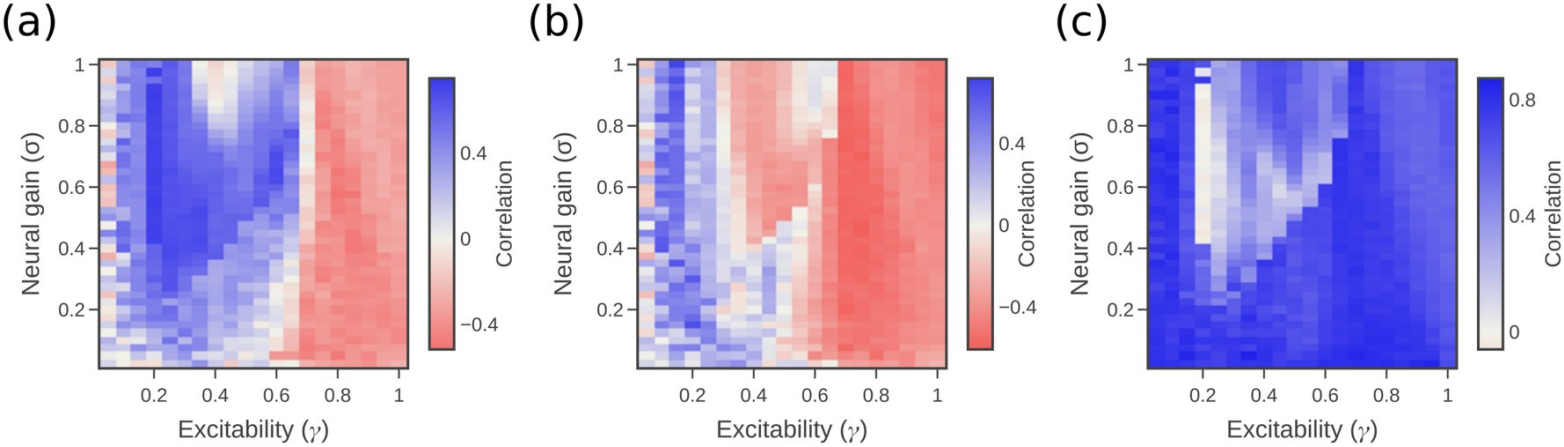
Correlations between information transfer and node degrees. (a) Correlation between TE rate and source in-degree. (b) Correlation between conditional TE rate and source in-degree. (c) Correlation between collective TE rate and target in-degree. Matching scale is used across all subfigures.

Fig 4a examines the correlation of the pairwise transfer entropy rate, averaged across all outgoing directed connections in the network for each source, with the in-degree of the source. This correlation is expected in general (having been observed in [57, 58] and somewhat related to [59–62]) because sources with higher in-degree have greater diversity of inputs, and so potentially more available information to transfer. The expected effect is observed in the supercritical phase, suggesting integration of the information from the different source inputs. Fig 4b shows the correlation of conditional transfer entropy rate to source in-degree (again with the conditional TE rate measure averaged for all outgoing connections for each source); the trend is far less pronounced there, only being observed around the critical boundary, due largely to the much smaller values of conditional TE than pairwise in Fig 3.

Fig 4c shows the correlation of the collective transfer entropy rate for each target to target in-degree. The target in-degree is used instead of the source because one value of collective transfer entropy is produced for each target, while the contribution over all sources is combined. Because the collective TE rate captures the combined effect from all sources, it can be expected that this will increase with the in-degree of the target, and so the correlation should be quite strong. This is observed across the phase space, though is weaker at the critical boundary (which on inspection appears to be due in part to larger non-linearities in the TE-degree relationship there).

### Inter-hemisphere information transfer is high in the supercritical region

**Fig 5:**
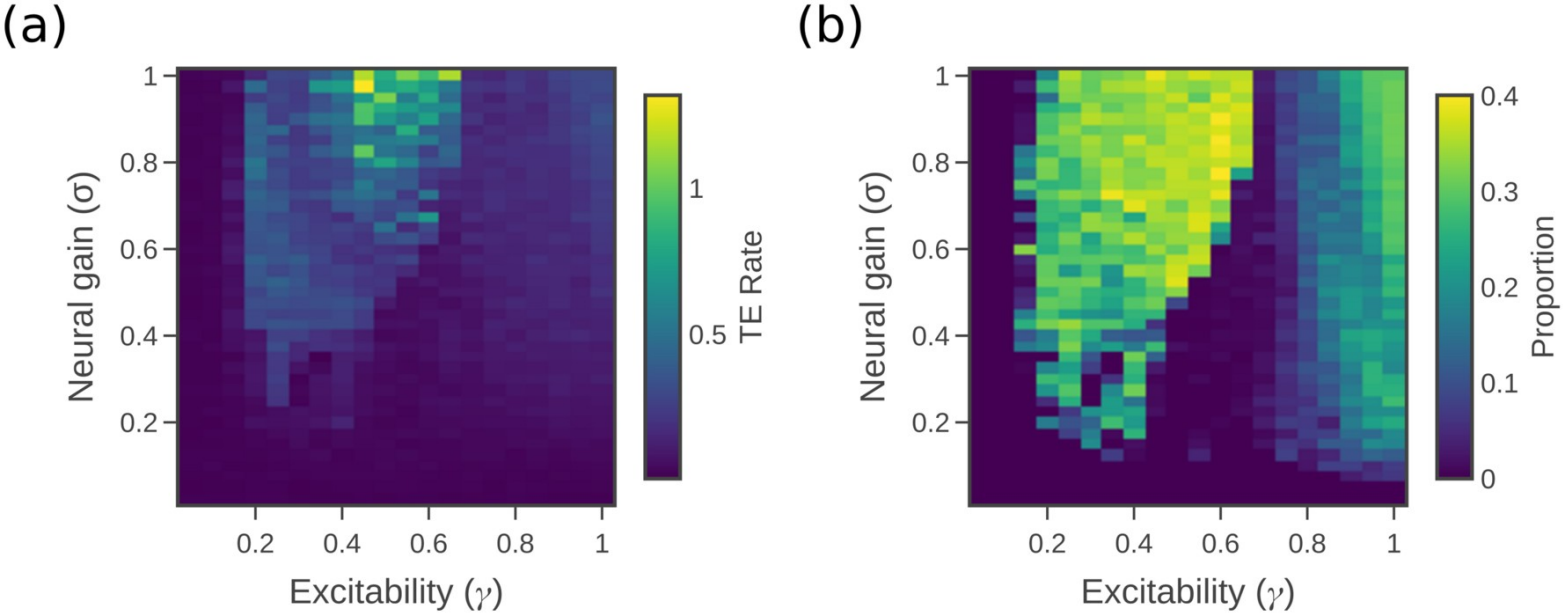
Information transfer between hemispheres. (a) TE rate over interhemisphere causal edges. (b) Proportion of significant TE rate measurements occuring between interhemisphere source and target.

The transfer entropy can be calculated solely for the causal edges which link regions between the two hemispheres. Only 38 links are inter-hemispheric (out of the total of 1494, not counting self loops). The weights of these connections are also relatively low, with an average weight of 1.07 (standard deviation of 0.86) compared to an average weight of 1.91 (standard deviation of 0.63) over all links, not counting self loops. Despite this, however, the average transfer entropy rate of these inter-hemisphere links in Fig 5a follows the same pattern as the standard pairwise transfer entropy rate seen in Fig 3a and the peak values are only 50% lower. This suggests that information transfer across hemispheres is significant, especially since Fig 5a favours the high *γ*, high *σ* part of the supercritical region, which may help explain why this trend is seen in Fig 3a.

Fig 5b shows the outcome of a second test which again highlights the importance of inter-hemisphere information transfer in the supercritical phase. This figure looks beyond causal links to compare the proportion of total statistically significant pairwise transfer entropy which occurs between hemispheres. The transfer entropy rate is first calculated for all pairwise combinations of source and target (whether they are linked in the directed connectome or not), at each point in the parameter space. However, pairs which do not give a level of transfer entropy statistically different to zero are ignored (see Methods). The remaining pairs are used to calculate the proportion of total pairwise transfer entropies that are accounted for by inter-hemisphere transfer, for each point in the parameter space. (Note that at points in *σ*, *γ* space where only a single pair had significant TE, the proportion is set to 0 in order to avoid noise in the plot where the proportion cannot be well determined). This proportion is close to half in the supercritical phase, showing that there is a large indirect effect of the information transferred between hemispheres. Even though there are only a few causal links between hemispheres, the information transferred by these “long” links is novel and becomes redistributed within the hemisphere, underpinning the higher levels of integration observed in the supercritical phase.

## Discussion

Using an information-theoretic decomposition, we extend previous work [7] by demonstrating that a gain-mediated phase transition in functional network topology is associated with a fundamental alteration in the information processing capacity of the whole brain network. Importantly, during this transition the underlying coupling strength and connectivity matrix are kept constant: the local dynamics are altered due to changes in the neural gain and excitability parameters, which then leads to changes in the effective connectivity (being a result of both local dynamics and large-scale structural connectivity). By modelling the distributed computation of the neural system in terms of information storage and information transfer, our results suggest that the shift from segregated to integrated states confers a computational alteration in the brain, which may be advantageous for certain cognitive tasks [18]. We thus reinterpret the gain-mediated transition in the functional configuration of the network in terms of the effective influence that neural regions can have over one another within a complex, adaptive, dynamical system [5]. Namely, subtle alterations in the neural gain control parameters lead to large transitions within the state space of functional topology, even within the constraints imposed by a hard-wired structural scaffold, with the resulting modulation of information processing capacity of the brain represented in different patterns of neural effective connectivity [1].

In previous work [7], we identified a distinct boundary that was mediated by alterations in neural gain parameters, which have long been linked to the functioning of the ascending (noradrenergic) arousal system [21]. Although we focus here on the noradrenergic system, recent work [63] has suggested that the effects of gain-mediated arousal in the brain may encompass a more distributed system of neuromodulatory nuclei. As such, the results of this study should be examined through this more distributed lens in future work. Extending this previous work (as currently modelled) into the domain of information processing, we here observed a qualitative shift in regional computational capacity on either side of the gain-mediated phase transition. Our information processing perspective models the interacting entities in the system as computational units, creating statistical models of how information is stored within or transferred between these entities during their intrinsic state updates [32, 33]. Here, we apply this perspective to study the information storage and transfer in the time-series activity of the membrane voltages *V*_*i*_ of each region, which interact as shown in (1)–(4). The qualitative shift in information processing we observed was that information storage (Fig 2a) was maximal in the subcritical region (at intermediate *γ*), whereas information transfer (Fig 3) was effectively non-existent in the subcritical region before it peaked in the supercritical region. This result is strongly aligned with the observed transition in phase synchrony observed in previous related studies. In a system of Kuramoto oscillators, Ceguerra et al. [57] showed that the synchronization process can be modelled as a distributed computation, with larger and increasing transfer entropy associated with more strongly synchronized or integrated network states. In the case of our model, we expect that maintaining synchronisation in the face of noise requires strong ongoing transfer between the relevant regions.

**Fig 6:**
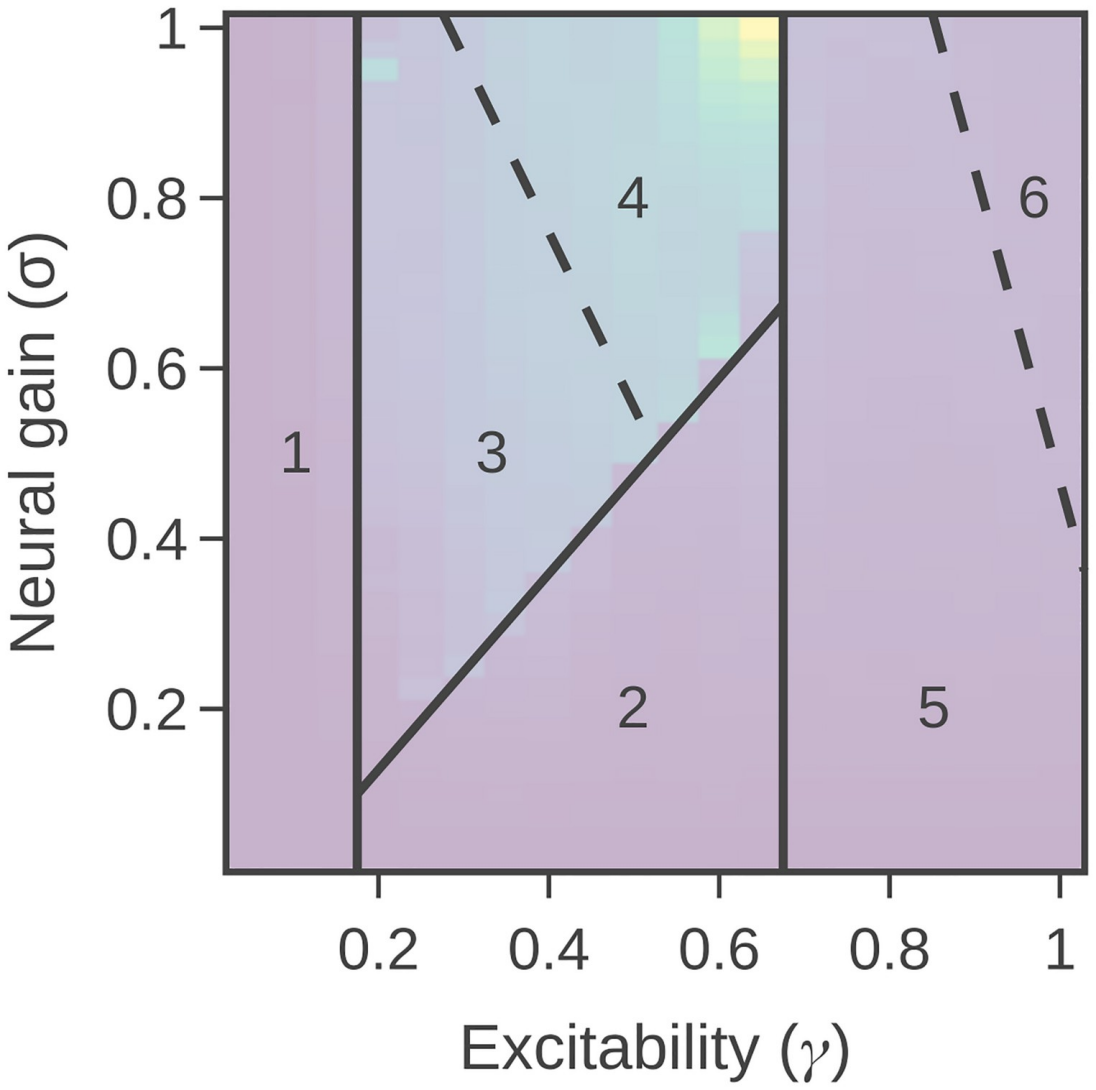
Phase portrait showing six identified regions. A transparent figure of the TE rate from Fig 3a is shown behind for comparison. Dotted lines represent a looser boundary, which are not observed in all measures.

Despite the strong qualitative effects observed at intermediate *γ*, the relationship between gain parameters and information processing was distinctly non-monotonic. By tracking information-theoretic measures across the parameter space, we were able to distinguish six unique zones with qualitative differences in information processing dynamics (Fig 6). For example, Zone 4 contained globally-synchronized oscillations which were relatively large, and also (compared to Zone 3) exhibited the strongest information transfer values. Note that this is not directly because the absolute range of the variables is larger (information measures on continuous-valued variables are scale independent [42]) but specifically due to variations in the relationships between dynamics of the regions. The differences between Zone 5 and Zone 6—both of which occur at high *γ* but have distinct between-hemispheric TE (TE6 ≫ TE5, Fig 5b) and AM rate (Fig 2a, including different dependencies on local network structure and self-loops in Fig 2e and 2f)—are also of interest, as they suggest that there may be distinct information processing signatures related to increasing multiplicative and response gain [64] to maximal levels, as in the case of epileptic seizures [65]. Future work should attempt to determine whether these categories are consistent across generative models, or perhaps relate to individual differences in topological recruitment across diverse cognitive tasks [17].

As a general framework for understanding distributed computation within complex systems, the translation of the previous results into the language of information storage and information transfer allows their comparison to other systems whose information dynamics have been shown to undergo phase transitions, including artificial neural networks [35], random Boolean networks [39, 40] as models for gene regulatory networks, the Ising model [37] and indeed Kuramoto oscillators [57] as mentioned above. There appears to be substantial universality among the results from these systems, with similar patterns of information storage and transfer often observed around critical phase transitions—and crucially these patterns are echoed here in transitions driven by alterations to neural gain parameters in our neural mass model. Across all of these systems, we consistently observe that dynamics of subcritical states are dominated by information storage operations underpinning higher segregation, whilst information transfer amongst the components of the networks plays a much more significant role in the dynamics of supercritical states leading to higher integration. In contrast to both, the critical state exhibits intermediate or strong values of both operations of information storage and transfer, achieving something of a balance in dynamics—a result which we emphasize was specifically observed again for the neural gain driven transitions examined here.

These insights allow us to address the question posed in our Introduction: are there computational advantages for the brain to operate in a near-critical state? In alignment with these results from other systems, and hypothesised as discussed earlier [13, 15, 16], the balance reached with both of these operations strongly exhibited near the critical state could be expected to support a wide range of general purpose cognitive tasks (requiring both types of operations), as well as in allowing flexibility for rapid transitions to either sub- or supercritical behaviour in order to alter the computational structure and dynamics as required. Indeed it is straightforward to identify situations that would require rapid transitions away from criticality toward more segregated or integrated operation. A relatively segregated, modular architecture is comprised of regions with high information storage, suggesting that situations in which a more segregated architecture is beneficial to cognitive performance—such as a motor-learning task [66] or visual vigilance [67]—may retain their capacity for improved performance by promoting heightened information storage. In contrast, cognitive states associated with integration—such as working memory [17] or attention [68, 69]—may reflect heightened inter-regional influence, and hence, information transfer between the diverse specialist regions housed within distinct locations in the cortex and subcortex [18]. The flexibility inherent in operating near a critical state would be expected to be crucial in supporting rapid transitions to support either broad type of task, and indeed the timescales that such transitions could be achieved in (near the critical state or otherwise) is an important area for future investigations.

The above interpretations align with a broader conjecture regarding utility of critical dynamics, such as in the “edge of chaos” hypothesis [31, 38] as well as more specific considerations regarding the utility of operating near criticality (but not directly at the “edge of chaos”) for the brain [13]. This convergence of results across the aforementioned systems suggests that the rules governing the organisation of whole brain dynamics may share crucial homology with other complex systems, in both biology and physics. However, inferring direct algorithmic correspondence will require more focused, direct comparison between the different systems. Furthermore, work remains to explain conditions leading to subtle differences in the patterns exhibited across the systems, for example the additional *maximization* of information storage and transfer capabilities near criticality in some transitions (e.g. Ising model [37]) but only a crossover without maximization in others (such as Kuramoto model [57] and the neural dynamics here).

The approach of information dynamics also provides a computational description of the dynamics of the system as they unfold at a local or point-wise level through time and across space [33]. Such descriptions provide quantitative insights regarding Marr’s “algorithmic” level [70] of how entities are represented within and operated on by a neural system [71]. In this study, we have not focused on the temporal dynamics of any particular task, but instead have examined the distribution of information processing signatures across the network. In particular, we have identified how the informational signature of brain dynamics relates to network structure as we transition across the neural gain parameter space. While the underlying network structure does not change, we seek to understand how its impact on the dynamics varies across the parameter space. Our approach allowed us to tease apart the relative importance of local network-supported versus internal mechanisms for information storage (Fig 2), where the local network support explained much of the storage (as suggested for different dynamics [51]) except for within the strongly segregated regime. We also found that source regions with large in-degrees (i.e. hubs) tended to be stronger information transfer sources, again for much of the parameter space except the strongly segregated regime. This aligned with our hypotheses and findings in other systems [57, 58], as well as related results such as that the degree of a node is correlated to the ratio of (average) outgoing to incoming information transfer from/to it in various dynamical models (including Ising network dynamics on the human connectome) [59, 60]. Finally, we compared the information transfer between hemispheres with the information transfer within hemisphere. Large proportions of information transfer could be apparently observed between non-directly linked regions across hemispheres in the supercritical regime, suggesting that the relatively large information transfer on the small number of inter-hemispheric causal edges supports significant global integration in this regime. These local views of network structure were thus linked to whole-brain macroscopic topology in important ways. The extent to which the patterns triggered by changes in neural gain parameters are targeted or global is a crucial question for future research, particularly given the recent appreciation of the heterogeneity of firing patterns within the locus coeruleus [72].

We note that the measures of information processing were estimated here using a Gaussian model, assuming linear interactions between the variables. This estimator was selected for efficient performance on the large data set and parameter space. Although such estimators may not directly capture strongly non-linear components of the interactions, they nevertheless provide a useful descriptive statistic even when the linear-Gaussian model is violated. We note that the linear component often dominates (e.g. [73]), and indeed the larger embeddings such estimators support provide additional terms to indirectly model non-linear components in AIS and TE.

In the present study, we simulated changes in population gain through two manipulations to a standard, symmetric sigmoid curve—modifying its height and changing its nonlinearity. The sigmoid-response curve is a first order approximation to the heterogenous and occasionally non-monotonic response curves of individual neurons and small-scale populations that rests upon the “diffusion approximation”—namely that more complex response curves at small scales merge into a homogenous sigmoid response curve at large-scale under the central-limit-theorem which holds as long as their states are only weakly correlated at large-scales [74–76]. In scenarios where this does not hold true, more complex effects—such as post-inhibitory rebound firing—can be accommodated by introducing asymmetries into the firing rate response curve, or adding additional dynamical variable, such as slow, voltage-dependent calcium states [77, 78]. These important effects are the subject of ongoing work in many groups, including our own.

The motivation for the previous study [7] was an attempt to explain the mechanistic basis of fluctuations in functional network topology that were observed in empirical BOLD data [17], which were hypothesized to be functionally related to ongoing dynamics in the ascending arousal system [17, 79]. However, the sluggish temporal nature of the haemodynamic response typically clouds the interpretation of causal or indeed effective connectivity between brain signals [80, 81]. In particular, variable delays between neural activity and peak haemodynamic response around the brain means that temporal precedence in the BOLD response does not necessarily imply neuronal causality. While approaches have been suggested to address this issue [82, 83], we instead investigated information-theoretic signatures on simulated neural data, which has a much higher effective sampling frequency than BOLD, and is also relatively unaffected by the temporal convolution that masks neural activity in the BOLD response. In doing so, we highlight important multi-level organisation within the simulated neural time series, in which whole-brain topological signatures (measured using BOLD) overlap with specific signatures of regional (neural) effective connectivity. It remains an open question whether this relationship holds in empirical data; increasing availability of intracranial human sEEG data will allow this to be tested more directly than with BOLD, however it bears mention that the direct comparison of empirical data with the results of simulations may in turn require the implementation of more biologically realistic models in order to capture the nuances present in biological data [84]. In any case, our approach certainly holds promise for advancing our interpretation of fluctuations in global network topology across cognitive states [18, 85, 86].

In conclusion, we have shown that modulating neural gain parameters in a biophysical model of brain dynamics leads to a shift in the computational signature of regional brain activity, in which the system shifts from a state dominated by self-referential information storage to one distinguished by significant inter-regional effective connectivity. These results provide a crucial algorithmic foundation for understanding the computational advantage of whole-brain network topological states, while simultaneously providing a plausible biological mechanism through which these changes could be instantiated in the brain—namely, alterations in the influence of the ascending arousal system over inter-regional connectivity.

## Materials and methods

### Simulation of neural activity

Neural activity was modelled (as per [7]) as a directed network of brain regions, with each region represented by an oscillating 2-dimensional neural mass model [45] derived by mode decomposition from the Fitzhugh-Nagumo single neuron model [87]. Directed coupling between 76 regions was derived from the CoCoMac connectome [88] with axonal time delays between regions computed from the length of fiber tracts estimated by diffusion spectrum imaging [47]. The model was simulated by stochastic Heun integration [89] using the open source framework The Virtual Brain [47].

The neural mass model at each region is given by the Langevin Eqs 1 and 2, which express the dynamics of local mean membrane potential (*V*) and the slow recovery variable (*W*) at each regional node *i*:

$$\begin{array}{c} {\dot{V}}_{i}\left(t\right)=20(W_{i}\left(t\right)+3V_{i}{\left(t\right)}^{2}-V_{i}{\left(t\right)}^{3}+\gamma I_{i})+\xi_{i}\left(t\right), \end{array}$$ (1)

$$\begin{array}{c} {\dot{W}}_{i}\left(t\right)=20\left(-W_{i}\left(t\right)-10V_{i}\left(t\right)\right)+\eta_{i}\left(t\right). \end{array}$$ (2)

Here, all *ξ*_*i*_ and *η*_*i*_ are independent standard Wiener noises, and *I* is the synaptic current, given by

$$\begin{array}{c} I_{i}=\sum_{j}A_{ij}S_{j}\left(t-\tau_{ij}\right), \end{array}$$ (3)

where *A*_*ij*_ is the connection weight from *j* to *i* in the directed connectivity matrix and *τ*_*ij*_ is the corresponding time delay from *j* to *i* (estimated as described above). Note that the network contains 1560 directed connections (66 being self-links), with *τ*_*ij*_ on non-self links having an average of 19.8 ms (standard deviation 8.32 ms). A sigmoid activation function was used to convert membrane potentials to normalized firing rates *S*_*i*_, where *m* = 1.5 was chosen to align the sigmoid with its typical input:

$$\begin{array}{c} S_{i}\left(t\right)=\frac{1}{1+e^{-\sigma (V_{i}\left(t\right)-m)}}. \end{array}$$ (4)

Using this model, we modulate the inter-regional coupling by varying the parameters for gain (*σ* in (4)) and excitability (*γ* in (1)) over a range of values between 0 and 1. At each parameter combination, membrane voltage (*V*_*i*_(*t*)) over time for each region was recorded as the time series input for the analysis of information dynamics. A sample length of 100,000 values per time series was used in the following analysis, corresponding to 50 seconds of one sample per 500 microseconds. The 2 kHz sampling rate was selected as described regarding the transfer entropy measure below. Each iteration was started from a different random initial condition.

Code implementing the model is freely available at https://github.com/macshine/gain_topology [90].

### Measures of information dynamics

The framework of information dynamics uses information-theoretic measures built on Shannon entropy to model the storage, transfer and modification of information within complex systems. It considers how the information in a variable *X*_*n*+1_ at time *n* + 1 can be modelled as being computed from samples of this and other processes at previous times. Information modelled as being contributed from the past of process *X* is labelled as information storage, while information modelled as contributed from other source processes *Y* is interpreted as information transfer.

The Java Information Dynamics Toolkit (JIDT) [91] was used to calculate these measures empirically using the time series of neuronal membrane voltage from the 76 regions. For each combination of *σ* and *γ* parameter values, the active memory rate was calculated for each region, and the transfer entropy rate was calculated for each combination of two regions. Collective and conditional transfer entropy rates were also calculated. Each of these measures is explained in the following sections. The linear-Gaussian estimator in JIDT was utilized in these calculations (which models the underlying processes as multivariate Gaussians with linear coupling). As per our Discussion, this remains a useful descriptive statistic even when the assumed model is violated.

#### Active information storage

Active Information Storage (AIS) [43] models the contribution of information storage to the dynamic state updates of a process *X* by measuring how much information from the past of *X* is observed in its next observation *X*_*n*+1_. It is defined as the expected mutual information between realizations $x_{n}^{\left(k,\tau \right)}$ of the past state $X_{n}^{\left(k,\tau \right)}$ at time *n* and the corresponding realizations *x*_*n*+1_ of the next value *X*_*n*+1_ of process X [43]:

$$\begin{array}{c} A_{X}\left(k,\tau \right)=I\left(X_{n}^{\left(k,\tau \right)};X_{n+1}\right). \end{array}$$ (5)

Formally, the states $x_{n}^{\left(k,\tau \right)}=\{x_{n-(k-1)\tau },\ldots ,x_{n-\tau },x_{n}\}$ are Takens’ embedding vectors [92] with embedding dimension *k* and embedding delay *τ*, which capture the underlying state of the process *X* for Markov processes of order *k*. In general, an embedding delay of *τ* ≥ 1 can be used, which may help to better empirically capture the state from a finite sample size. (Note that non-uniform embeddings can be used [93]).

The determination of these embedding parameters followed the method of Garland et al. [94] finding the values which maximize the AIS, with the important additional inclusion of bias correction (because increasing *k* generally serves to increase bias of the estimate) [95]. For several sample *σ*, *γ* pairs in both the sub- and supercritical regimes we examined these parameter choices across all regions (up to *k*, *τ* ≤ 30), and found the optimal choices to be consistently close to *k* = 25 and *τ* = 12 for all variables, for the sampling interval Δ*t* = 0.5 ms with 10000 samples. For example, for *σ* = 0.3, *γ* = 0.5 (subcritical), the mean of the optimal *k* across regions was 23.8, with standard deviation 6.7, and median 25; whilst the optimal *τ* across regions was 13.6 with standard deviation 4.8 and median 12. Similarly for *σ* = 0.6, *γ* = 0.5 (supercritical), the mean of the optimal *k* across regions was 24.1, with standard deviation 3.2, and median 25; whilst the optimal *τ* across regions was 13.1 with standard deviation 5.3 and median 13. As such, *k* = 25 and *τ* = 12 were used for all investigations. The total period of history covered by this embedding (300 time points, or 150 ms) corresponds to approximately 1.5 periods of the underlying oscillations in the subcritical regime and slightly under 1 period in the supercritical regime.

Note that while a larger AIS is likely to give rise to a larger auto-correlation time, there are significant differences between the two measures which make AIS much more powerful, and directly relevant for modelling the utilisation of information storage (unlike autocorrelation). Primarily these differences stem from AIS examining the relationship between multiple past values (as the embedded past) to the current value of the time series, taking into consideration whether those past values are providing the same information redundantly or unique information, or indeed are synergistically providing more when they are considered together. Auto-correlation values in contrast only ever examine relationships from one past value to the current, and are unable to resolve such complexities in the process. (As an information-theoretic measure, AIS can also capture non-linear interactions, although in this study we only use a linear estimator). This leads to the AIS providing very different values to auto-correlation, and indeed much richer insights. For example, significant reductions were observed in AIS in multiple regions of Autism Spectrum Disorder (ASD) subjects versus controls [96], indicating significantly reduced use or precision of priors in dynamic state updates of ASD subjects. In contrast, no such differences were observable using auto-correlation times or signal power.

Because of the fast sample rate (Δ*t* = 0.5 ms) of the neuronal time series, proper analysis requires using a formulation of the information-theoretic measures suitable for continuous time processes. In general, this means that information storage and information transfer are conceptualized as measures that *accumulate* over some finite time interval at an associated *rate* [48]. Both the accumulated and rate measures, however, diverge in the limit as the time step approaches zero. Intuitively, for continuous processes such as those here, this is because all information about the next time step can be captured by the previous time step in the limit as the two samples become essentially identical. These divergent properties can be circumvented by decomposing active information storage into components, $A_{X}=I_{X}+{\dot{M}}_{X}\Delta t+O\left({\Delta t}^{2}\right)$, comprising [48]:

- the instantaneous predictive capacity which measures the information storage from the immediately previous time step, *I*_*X*_ = *I*(*X*_*n*_; *X*_*n*+1_), and
- the **active memory utilisation rate** (AM rate) which measures the additional accumulation rate of information storage from time steps before that, ${\dot{M}}_{X}=I\left(X_{n-1}^{\left(k-1\right)};X_{n+1}|X_{n}\right)/\Delta t$.

The instantaneous predictive capacity inherits the divergent nature of the active information storage, while the active memory utilization rate takes on the intuitive representation of memory as a rate [48]. Crucially, ${\dot{M}}_{X}$ converges to a limiting value as Δ*t* → 0 for well-behaved continuous processes such as those considered here unlike *A*_*X*_ and *I*_*X*_ (see full details in [48]), and thus only ${\dot{M}}_{X}$ is used in our investigations here. As a rate, the units of measurement of ${\dot{M}}_{X}$ are in bits per second. As recommended by Spinney et al. [48, 52], Δ*t* = 0.5 ms was selected on confirming that the transfer entropy rate (see next subsection) and ${\dot{M}}_{X}$ are stable to Δ*t* in this regime and appear to have converged to a limiting value as Δ*t* → 0.

#### Transfer entropy

Transfer entropy [44, 97] models the contribution of information transfer from a source process *Y* to the dynamic state updates of a destination (or target) process *X* by measuring the amount of information that *Y* provides about the next state of process *X* in the context of the destination’s past. This perspective of modelling of information transfer after first considering storage from the past contrasts the two operations, and ensures that no information storage is attributed as having been transferred [97]. The transfer entropy has been used to model information flow from neural time-series recordings, using various recording types and experiments, e.g. [98–103].

Quantitatively, the transfer entropy is the expected mutual information from realizations $y_{n-u+1}^{\left(l,\omega \right)}$ of the state $Y_{n-u+1}^{\left(l,\omega \right)}$ of a source process *Y* over a delay *u* to the corresponding realizations *x*_*n*+1_ of the next value *X*_*n*+1_ of the destination process *X*, conditioned on realizations $x_{n}^{\left(k,\tau \right)}$ of its previous state $X_{n}^{\left(k,\tau \right)}$:

$$\begin{array}{c} T_{Y\to X}\left(k,\tau ,l,\omega ,u\right)=I\left(Y_{n-u+1}^{\left(l,\omega \right)};X_{n+1}|X_{n}^{\left(k,\tau \right)}\right) \end{array}$$ (6)

The target embedding parameters *k* and *τ* are set to the same values as determined for the previous information storage calculations, as is standard when the two operations are being considered. In general, an embedding of the source state $y_{n-u+1}^{\left(l,\omega \right)}$ with *l* > 1 could be considered as this would allow *Y* to be a Markovian process where multiple past values of *Y* in addition to *y*_*n*−*u*+1_ are information sources to *x*_*n*+1_. However for this analysis only *l* = 1 previous time step of the source process is used (denoted *T*_*Y*→*X*_(*k*, *τ*, *u*)), in line with the known contribution of a single value of the source time series to the target in the neural model in (1)–(4) (as is standard in this situation [33]).

In order to best model information transfer, the source processes for TE measurements are constrained to the known causal information contributors [53]. In this case these are the upstream parents of the target in the structural connectivity matrix. Further, the source-target delays *u* are set to the number of discrete time steps aligning with (or rather, being the smallest integer of discrete steps giving a time delay larger than) the known source-target delays used in the model (3), as is best practice [33, 54]. Where, the TE is estimated for pairs that are not directly causally linked in the model, the time delay is estimated in the same way from corresponding fiber tract lengths.

As mentioned in the previous subsection, the small time steps of the neuronal time series requires us to consider continuous-time formulations, meaning we compute a **transfer entropy rate**, ${\dot{T}}_{Y\to X}\left(k,\tau ,u\right)=T_{Y\to X}\left(k,\tau ,u\right)/\Delta t$ [48, 52].

The use of the linear-Gaussian estimator in JIDT for TE estimation makes the calculated transfer entropy (rate) equivalent, up to a constant, to Granger causality (rate) [104, 105].

Finally, note that transfer entropy estimations can be non-zero even where the source and destination processes have no directional relationship, due to estimator variance and bias (see summary in [97, Sec. 4.5.1]). As such, one can make a statistical test of whether a transfer entropy estimate is statistically different from the null distribution of values that would be observed for source and destination processes with similar properties but no directed relationship. As described in [91, App. A.5], the null transfer entropies are created from surrogate time series generated by permutation resampling of the source embeddings $y_{n-u+1}^{\left(l,\omega \right)}$: these retain the memory in the target $p(x_{n+1}|x_{n}^{\left(k,\tau \right)})$ and the source distribution p($y_{n-u+1}^{\left(l,\omega \right)}$) but not the source-target transition relationship $p(x_{n+1}|x_{n}^{\left(k,\tau \right)},y_{n-u+1}^{\left(l,\omega \right)})$. We perform a test of statistical significance for each directed pair of processes in producing Fig 5b, retaining there only the transfer entropies for pairs that were determined to be statistically significant against the null distribution against a p-value threshold of *α* = 0.05. This test is carried out analytically for the Gaussian estimator, as per [37] (summarised in [91, App. A.5]), with a Bonferroni correction for all directed pairs that are tested.

#### Conditional and collective transfer entropy

Higher order terms of information transfer can capture the multivariate effects from multiple sources to a single target. Two higher order terms which were calculated are the conditional and collective transfer entropies.

Conditional transfer entropy [55, 56, 106] extends the basic form of transfer entropy by conditioning on the history of another source process, *Z*. This captures the mutual information between the past of source *Y* and the next value of target *X*, conditioned on both the history $X_{n}^{\left(k,\tau \right)}$ of *X* and the history of conditional source *Z*:

$$\begin{array}{c} T_{Y\to X|Z}\left(k,\tau ,u\right)=I\left(Y_{n-u+1};X_{n+1}|X_{n}^{\left(k,\tau \right)},Z_{n}\right) \end{array}$$ (7)

Of course, the above may in general incorporate embeddings for both *Y* and *Z*, and a delay *Z* to *X*, and can be extended to condition on several other sources **Z** (excluding *Y*) at once. It should be noted that a conditioned transfer entropy can be either larger or smaller than the unconditioned measure, in the same way that a conditional mutual information can both increase due to the addition of synergistic information that can only be decoded with knowledge of both the source and conditional, as well as decrease due to a removal of redundant information provided by both the source and conditional. The conditional transfer entropy thus includes unique information from the source but not the conditional, and synergistic information provided by the source and conditional together, in the context of the past of the target [55, 107]. These components cannot be pulled apart using the tools of traditional information theory, but efforts are being made by approaches of Partial Information Decomposition (PID) [108–110].

At the same time, collective transfer entropy [56, 100] models the total information transfer from a set of sources to a target, capturing unique information from each source, avoiding double-counting redundant information across the sources, and capturing multivariate synergistic effects. Given a multivariate set of sources **Y**, this refers to the measure *T*_**Y**→*X*_(*k*, *τ*, *u*) (again ignoring possible embeddings on the **Y** processes, or different delays for each *Y* in **Y**).

For these experiments, we compute conditional transfer entropy rate and collective transfer entropy rate, similar to the pairwise transfer entropy rate. Only the highest order conditional transfer entropy is calculated (known specifically as complete transfer entropy [55, 56]). This means that for each causal source, the conditional transfer entropy to a particular target involved conditioning on *all* the other causal sources to that target, as identified from the directed connectome. Also, we calculated the collective transfer entropy to a given target from all causal sources to that target region, as identified from the directed connectome. The delays from each source to the target, as well as for each conditional source to the target, are determined so as to match those for the model in (3) as per the pairwise transfer entropy above.

### Network motifs

The 76 regions of this model are connected by a directed, weighted network *A*_*ij*_ (see (3)) derived from the CoCoMac connectome [88]. The information storage of each region is expected to be related to both the strength of self-loops internal to that region as well as distributed network effects, since both intuitively provide mechanisms for the past activity of a region to influence its future dynamics [49, 50]. The support for storage provided by network effects will depend on the number (and weight) of certain network motifs which provide feedforward and feedback loops involving that region. For linearly-coupled Gaussian processes the active information storage can be calculated as a function of weighted counts of these motifs [51]. However, for the more complex dynamics of this system involving non-linearities in (1) and (4), we cannot derive an exact relationship. Instead, we generate heuristics to approximate the relative weights of self-loops and (relevant parts of the) distributed network structure in supporting storage at a particular node or region *a*, in comparison to all other regions. Next, we correlate (via Pearson correlation) these heuristics for each region to their active memory, at each point in the parameter space (across all nodes for a given *σ*, *γ*), in order to infer which mechanism (local or network effects) appears to be a more relevant factor in supporting information storage across the network at each point in the parameter space. Details of these heuristics are as follows.

We approximate the relative capacity of network structure for information storage for a given region *a* (in comparison to other regions) in the **local network support** heuristic, which is a weighted linear combination of certain motif counts involving *a*. As above, we cannot derive the precise capacity for information storage provided by these motifs under these dynamics, so our heuristic simply counts the (weighted) number of such motifs that are known to be relevant in general for information storage at node *a*. The motifs which were considered (based on those identified in [51]) incorporated feedback loops including the target node *a* and feedforward loops terminating at node *a* (see Fig 2d). The weighting given to each motif depends on the number of incoming links to *a* and their edge weights (which are derived from the coupling strengths *A*_*ij*_). First the edge weight of each link is normalized by the total incoming edge weight for each target (excluding self loops) to generate *C* = *D*^−1^(*A* − diag(*A*_1,1_, *A*_2,2_, …, *A*_76,76_)), where *D* = diag(*d*_1_, *d*_2_…, *d*_76_) with *d*_*i*_ = ∑_*j*≠*i*_ *A*_*ij*_. This normalization takes inspiration from generation of normalized Laplacians [111], and is intended to weight the contribution along each edge on a path as relative to other contributions into the same target node (i.e. the more incoming edges one edge competes with, the smaller its relative contribution to the target’s dynamics will be). The local network support Ψ_*a*_ at node *a* is then computed as a linear sum of the relevant motifs at node *a* using the normalized weights *C*:

$$\begin{array}{c} \Psi_{a}=\sum_{b}C_{ba}C_{ab}+\sum_{bc}C_{ba}C_{cb}C_{ac}+\sum_{bc}C_{bc}C_{ab}C_{ac}+\sum_{bcd}C_{cd}C_{bc}C_{ab}C_{ad}. \end{array}$$ (8)

Note that the four weighted motif counts in the equation for Ψ_*a*_ correspond respectively to the four motifs shown in Fig 2d to contribute to storage in the dynamics of node *a*. The contribution from longer motifs diminishes with length due to the normalisation, and so we limit (8) to the shortest two contributing feedback and feedfoward motifs (except for any self-loop at *a*). We emphasise again that the heuristic Ψ_*a*_ does not precisely capture the extent to which information storage is supported by the local network structure at node *a*; by simply counting the (weighted) relevant motifs for storage at *a* it is intended to approximately indicate relative capacity provided for network-supported information storage at different parts of the system.

As described above, the self-loops were ignored in the local network support measure in order to consider relative support for memory from distributed network effects only in Ψ_*a*_. The relative contribution of self-loops *A*_*ii*_ (i.e. synaptic connections between neurons within the same brain region) to memory was instead analysed separately so as to compare the two effects. A similar weighting was applied to self loops in order to normalize their contribution with respect to total incoming edge weights (this time including the self loop). Here, we first computed *F* = *G*^−1^*A*, where *G* = diag(*g*_1_, *g*_2_…, *g*_76_), *g*_*i*_ = ∑_*j*_ *A*_*ij*_. Then, in order to evaluate the relative strength of contribution of self-loops to memory across the parameter space, we correlated the *F*_*ii*_ with the observed active memory rate across all nodes for each given *σ*, *γ* pair. Note that we focus here on the synaptic connections between neurons within the same brain region modelled by *A*_*ii*_, which is mediated by the neural gain parameters. This analysis does not include the feedback terms in *V* and *W* in (1) and (2) which correspond to self-coupling in the oscillatory dynamics of individual neurons that make up the population. We do not include those terms because they are constant across regions and are not moderated by the neural gain parameters.

## References

[1] Friston KJ. Functional and effective connectivity: a review. Brain connectivity. 2011;1(1):13–36. 10.1089/brain.2011.0008 22432952

[2] Breakspear M. Dynamic models of large-scale brain activity. Nature Neuroscience. 2017;20(3):340 10.1038/nn.4497 28230845

[3] Swanson LW. Brain architecture: understanding the basic plan. Oxford University Press; 2012.

[4] Bullmore E, Sporns O. Complex brain networks: graph theoretical analysis of structural and functional systems. Nature Reviews Neuroscience. 2009;10:186 10.1038/nrn2575 19190637

[5] Varela F, Lachaux JP, Rodriguez E, Martinerie J. The brainweb: Phase synchronization and large-scale integration. Nature Reviews Neuroscience. 2001;2:229 10.1038/35067550 11283746

[6] Deisboeck TS, Kresh JY. Complex Systems Science in Biomedicine. Boston, MA: Springer Inc; 2006.

[7] Shine JM, Aburn MJ, Breakspear M, Poldrack RA. The modulation of neural gain facilitates a transition between functional segregation and integration in the brain. eLife. 2018;7:e31130 10.7554/eLife.31130 29376825 PMC5818252

[8] Cocchi L, Gollo LL, Zalesky A, Breakspear M. Criticality in the brain: A synthesis of neurobiology, models and cognition. Progress in Neurobiology. 2017;158:132–152. 10.1016/j.pneurobio.2017.07.002 28734836

[9] Beggs JM, Plenz D. Neuronal avalanches in neocortical circuits. Journal of Neuroscience. 2003;23(35):11167–11177. 10.1523/JNEUROSCI.23-35-11167.2003 14657176 PMC6741045

[10] Shew WL, Yang H, Yu S, Roy R, Plenz D. Information capacity and transmission are maximized in balanced cortical networks with neuronal avalanches. Journal of Neuroscience. 2011;31(1):55–63. 10.1523/JNEUROSCI.4637-10.2011 21209189 PMC3082868

[11] Priesemann V, Wibral M, Valderrama M, Pröpper R, Le Van Quyen M, Geisel T, et al Spike avalanches in vivo suggest a driven, slightly subcritical brain state. Frontiers in Systems Neuroscience. 2014;8(108). 10.3389/fnsys.2014.00108 25009473 PMC4068003

[12] Priesemann V, Valderrama M, Wibral M, Le Van Quyen M. Neuronal Avalanches Differ from Wakefulness to Deep Sleep—Evidence from Intracranial Depth Recordings in Humans. PLOS Computational Biology. 2013;9(3):e1002985 10.1371/journal.pcbi.1002985 23555220 PMC3605058

[13] Wilting J, Dehning J, Neto JP, Rudelt L, Wibral M, Zierenberg J, et al. Dynamic Adaptive Computation: Tuning network states to task requirements. arXiv preprint arXiv:180907550. 2018;. 10.3389/fnsys.2018.00055 PMC6232511 30459567

[14] Wilting J, Priesemann V. Inferring collective dynamical states from widely unobserved systems. Nature Communications. 2018;9(1):2325 10.1038/s41467-018-04725-4 29899335 PMC5998151

[15] Deco G, Jirsa VK. Ongoing Cortical Activity at Rest: Criticality, Multistability, and Ghost Attractors. Journal of Neuroscience. 2012;32(10):3366–3375. 10.1523/JNEUROSCI.2523-11.2012 22399758 PMC6621046

[16] Hahn G, Ponce-Alvarez A, Monier C, Benvenuti G, Kumar A, Chavane F, et al Spontaneous cortical activity is transiently poised close to criticality. PLOS Computational Biology. 2017;13(5):1–29. 10.1371/journal.pcbi.1005543 PMC5464673 28542191

[17] Shine JM, Bissett PG, Bell PT, Koyejo O, Balsters JH, Gorgolewski KJ, et al The Dynamics of Functional Brain Networks: Integrated Network States during Cognitive Task Performance. Neuron. 2016;92(2):544–554. 10.1016/j.neuron.2016.09.018 27693256 PMC5073034

[18] Shine JM, Poldrack RA. Principles of dynamic network reconfiguration across diverse brain states. NeuroImage. 2017;180:396–405. 10.1016/j.neuroimage.2017.08.010 28782684

[19] Lee SH, Dan Y. Neuromodulation of Brain States. Neuron. 2012;76(1):209–222. 10.1016/j.neuron.2012.09.012 23040816 PMC3579548

[20] Marder E. Neuromodulation of Neuronal Circuits: Back to the Future. Neuron. 2012;76(1):1–11. 10.1016/j.neuron.2012.09.010 23040802 PMC3482119

[21] Servan-Schreiber D, Printz H, Cohen J. A network model of catecholamine effects: gain, signal-to-noise ratio, and behavior. Science. 1990;249(4971):892–895. 2392679 10.1126/science.2392679

[22] Fazlali Z, Ranjbar-Slamloo Y, Adibi M, Arabzadeh E. Correlation between Cortical State and Locus Coeruleus Activity: Implications for Sensory Coding in Rat Barrel Cortex. Frontiers in Neural Circuits. 2016;10:14 10.3389/fncir.2016.00014 27047339 PMC4805600

[23] Beggs J, Timme N. Being Critical of Criticality in the Brain. Frontiers in Physiology. 2012;3(163). 10.3389/fphys.2012.00163 22701101 PMC3369250

[24] Chialvo DR. Emergent complex neural dynamics. Nature Physics. 2010;6:744 10.1038/nphys1803

[25] Scheffer M, Bascompte J, Brock WA, Brovkin V, Carpenter SR, Dakos V, et al Early-warning signals for critical transitions. Nature. 2009;461(7260):53–59. 10.1038/nature08227 19727193

[26] Kuehn C. A mathematical framework for critical transitions: Bifurcations, fast–slow systems and stochastic dynamics. Physica D: Nonlinear Phenomena. 2011;240(12):1020–1035. 10.1016/j.physd.2011.02.012

[27] Aburn MJ, Holmes CA, Roberts JA, Boonstra TW, Breakspear M. Critical Fluctuations in Cortical Models Near Instability. Frontiers in Physiology. 2012;3:331 10.3389/fphys.2012.00331 22952464 PMC3424523

[28] Matsuda H, Kudo K, Nakamura R, Yamakawa O, Murata T. Mutual information of ising systems. International Journal of Theoretical Physics. 1996;35(4):839–845. 10.1007/BF02330576

[29] Ribeiro AS, Kauffman SA, Lloyd-Price J, Samuelsson B, Socolar JES. Mutual information in random Boolean models of regulatory networks. Physical Review E. 2008;77(1):011901 10.1103/PhysRevE.77.011901 18351870

[30] Prokopenko M, Lizier JT, Obst O, Wang XR. Relating Fisher information to order parameters. Physical Review E. 2011;84:041116 10.1103/PhysRevE.84.041116 22181096

[31] Langton CG. Computation at the edge of chaos: Phase transitions and emergent computation. Physica D: Nonlinear Phenomena. 1990;42(1):12–37. 10.1016/0167-2789(90)90064-V

[32] Lizier JT. The Local Information Dynamics of Distributed Computation in Complex Systems. Berlin/Heidelberg: Springer; 2013.

[33] Lizier JT. In: Wibral M, Vicente R, Lizier JT, editors. Measuring the Dynamics of Information Processing on a Local Scale in Time and Space. Berlin/Heidelberg: Springer; 2014 p. 161–193.

[34] Priesemann V, Lizier J, Wibral M, Bullmore ET, Paulsen O, Charlesworth P, et al Self-organization of information processing in developing neuronal networks. BMC Neuroscience. 2015;16(Suppl 1):P221+. 10.1186/1471-2202-16-S1-P221

[35] Boedecker J, Obst O, Lizier JT, Mayer NM, Asada M. Information processing in echo state networks at the edge of chaos. Theory in Biosciences. 2012;131(3):205–213. 10.1007/s12064-011-0146-8 22147532

[36] Bertschinger N, Natschläger T. Real-Time Computation at the Edge of Chaos in Recurrent Neural Networks. Neural Computation. 2004;16(7):1413–1436. 10.1162/089976604323057443 15165396

[37] Barnett L, Lizier JT, Harré M, Seth AK, Bossomaier T. Information Flow in a Kinetic Ising Model Peaks in the Disordered Phase. Physical Review Letters. 2013;111(17):177203 10.1103/PhysRevLett.111.177203 24206517

[38] Kauffman SA. The Origins of Order: Self-Organization and Selection in Evolution. New York: Oxford University Press; 1993.

[39] Lizier JT, Pritam S, Prokopenko M. Information dynamics in small-world boolean networks. Artificial Life. 2011;17(4):293–314. 10.1162/artl_a_00040 21762020

[40] Lizier JT, Prokopenko M, Zomaya AY. The information dynamics of phase transitions in random Boolean networks. In: Bullock S, Noble J, Watson R, Bedau MA, editors. Proceedings of the Eleventh International Conference on the Simulation and Synthesis of Living Systems (ALife XI), Winchester, UK. Cambridge, MA: MIT Press; 2008. p. 374–381.

[41] Watts DJ, Strogatz SH. Collective dynamics of ‘small-world’ networks. Nature. 1998;393(6684):440 10.1038/30918 9623998

[42] Cover TM, Thomas JA. Elements Of Information Theory 2nd Ed Wiley; 2006.

[43] Lizier JT, Prokopenko M, Zomaya AY. Local measures of information storage in complex distributed computation. Information Sciences. 2012;208:39–54. 10.1016/j.ins.2012.04.016

[44] Schreiber T. Measuring information transfer. Physical Review Letters. 2000;85(2):461 10.1103/PhysRevLett.85.461 10991308

[45] Stefanescu RA, Jirsa VK. Reduced representations of heterogeneous mixed neural networks with synaptic coupling. Physical Review E. 2011;83(2):026204 10.1103/PhysRevE.83.026204 21405893

[46] Bakker R, Wachtler T, Diesmann M. CoCoMac 2.0 and the future of tract-tracing databases. Frontiers in Neuroinformatics. 2012;6:30 10.3389/fninf.2012.00030 23293600 PMC3530798

[47] Sanz Leon P, Knock S, Woodman M, Domide L, Mersmann J, McIntosh A, et al The Virtual Brain: a simulator of primate brain network dynamics. Frontiers in Neuroinformatics. 2013;7(10). 10.3389/fninf.2013.00010 23781198 PMC3678125

[48] Spinney RE, Lizier JT. Characterizing information-theoretic storage and transfer in continuous time processes. Physical Review E. 2018;98(1):012314 10.1103/PhysRevE.98.012314 30110808

[49] Wibral M, Lizier JT, Vögler S, Priesemann V, Galuske R. Local active information storage as a tool to understand distributed neural information processing. Frontiers in Neuroinformatics. 2014;8:1 10.3389/fninf.2014.00001 24501593 PMC3904075

[50] Zipser D, Kehoe B, Littlewort G, Fuster J. A spiking network model of short-term active memory. The Journal of Neuroscience. 1993;13(8):3406–3420. 10.1523/JNEUROSCI.13-08-03406.1993 8340815 PMC6576542

[51] Lizier JT, Atay FM, Jost J. Information storage, loop motifs, and clustered structure in complex networks. Physical Review E. 2012;86(2):026110 10.1103/PhysRevE.86.026110 23005828

[52] Spinney RE, Prokopenko M, Lizier JT. Transfer entropy in continuous time, with applications to jump and neural spiking processes. Physical Review E. 2017;95(3):032319 10.1103/PhysRevE.95.032319 28415203

[53] Lizier JT, Prokopenko M. Differentiating information transfer and causal effect. The European Physical Journal B. 2010;73(4):605–615. 10.1140/epjb/e2010-00034-5

[54] Wibral M, Pampu N, Priesemann V, Siebenhühner F, Seiwert H, Lindner M, et al Measuring Information-Transfer Delays. PLoS ONE. 2013;8(2):e55809 10.1371/journal.pone.0055809 23468850 PMC3585400

[55] Lizier JT, Prokopenko M, Zomaya AY. Local information transfer as a spatiotemporal filter for complex systems. Phys Rev E. 2008;77:026110 10.1103/PhysRevE.77.026110 18352093

[56] Lizier JT, Prokopenko M, Zomaya AY. Information modification and particle collisions in distributed computation. Chaos: An Interdisciplinary Journal of Nonlinear Science. 2010;20(3):037109 10.1063/1.3486801 20887075

[57] Ceguerra RV, Lizier JT, Zomaya AY. Information storage and transfer in the synchronization process in locally-connected networks. In: 2011 IEEE Symposium on Artificial Life (ALIFE); 2011. p. 54–61.

[58] Lizier JT, Prokopenko M, Cornforth DJ. The information dynamics of cascading failures in energy networks. In: Proceedings of the European Conference on Complex Systems (ECCS); 2009. p. 54+.

[59] Marinazzo D, Pellicoro M, Wu G, Angelini L, Cortés JM, Stramaglia S. Information Transfer and Criticality in the Ising Model on the Human Connectome. PLoS ONE. 2014;9(4):e93616 10.1371/journal.pone.0093616 24705627 PMC3976308

[60] Marinazzo D, Wu G, Pellicoro M, Angelini L, Stramaglia S. Information flow in networks and the law of diminishing marginal returns: evidence from modeling and human electroencephalographic recordings. PLoS ONE. 2012;7(9):e45026 10.1371/journal.pone.0045026 23028745 PMC3445562

[61] Timme NM, Ito S, Myroshnychenko M, Nigam S, Shimono M, Yeh FC, et al High-Degree Neurons Feed Cortical Computations. PLOS Computational Biology. 2016;12(5):e1004858 10.1371/journal.pcbi.1004858 27159884 PMC4861348

[62] Faber SP, Timme NM, Beggs JM, Newman EL. Computation is concentrated in rich clubs of local cortical networks. Network Neuroscience. 2019;3(2):384–404. 10.1162/netn_a_00069 30793088 PMC6370472

[63] Lovett-Barron M, Andalman AS, Allen WE, Vesuna S, Kauvar I, Burns VM, et al Ancestral circuits for the coordinated modulation of brain state. Cell. 2017;171(6):1411–1423. 10.1016/j.cell.2017.10.021 29103613 PMC5725395

[64] Thiele A, Bellgrove MA. Neuromodulation of Attention. Neuron. 2018;97(4):769–785. 10.1016/j.neuron.2018.01.008 29470969 PMC6204752

[65] Pietersen ANJ, Cheong SK, Munn B, Gong P, Martin PR, Solomon SG. Relationship between cortical state and spiking activity in the lateral geniculate nucleus of marmosets. The Journal of Physiology. 2017;595(13):4475–4492. 10.1113/JP273569 28116750 PMC5491878

[66] Bassett DS, Wymbs NF, Porter MA, Mucha PJ, Carlson JM, Grafton ST. Dynamic reconfiguration of human brain networks during learning. Proceedings of the National Academy of Sciences of the United States of America. 2011;108(18):7641–7646. 10.1073/pnas.1018985108 21502525 PMC3088578

[67] Sadaghiani S, Poline JB, Kleinschmidt A, D’Esposito M. Ongoing dynamics in large-scale functional connectivity predict perception. Proceedings of the National Academy of Sciences. 2015;112(27):8463–8468. 10.1073/pnas.1420687112 PMC4500238 26106164

[68] Shine JM, Koyejo O, Poldrack RA. Temporal metastates are associated with differential patterns of time-resolved connectivity, network topology, and attention. Proceedings of the National Academy of Sciences. 2016;113(35):9888–9891. 10.1073/pnas.1604898113 PMC5024627 27528672

[69] Ekman M, Derrfuss J, Tittgemeyer M, Fiebach CJ. Predicting errors from reconfiguration patterns in human brain networks. Proceedings of the National Academy of Sciences. 2012;109(41):16714–16719. 10.1073/pnas.1207523109 PMC3478635 23012417

[70] Marr D. Vision: A Computational Investigation into the Human Representation and Processing of Visual Information. New York, NY, USA: Henry Holt and Co., Inc.; 1982.

[71] Wibral M, Lizier JT, Priesemann V. Bits from brains for biologically inspired computing. Frontiers in Robotics and AI. 2015;2:5 10.3389/frobt.2015.00005

[72] Totah NK, Neves RM, Panzeri S, Logothetis NK, Eschenko O. The Locus Coeruleus Is a Complex and Differentiated Neuromodulatory System. Neuron. 2018;99(5):1055–1068.e6. 10.1016/j.neuron.2018.07.037 30122373

[73] Hlinka J, Paluš M, Vejmelka M, Mantini D, Corbetta M. Functional connectivity in resting-state fMRI: Is linear correlation sufficient? NeuroImage. 2011;54(3):2218–2225. 10.1016/j.neuroimage.2010.08.042 20800096 PMC4139498

[74] Marreiros AC, Daunizeau J, Kiebel SJ, Friston KJ. Population dynamics: Variance and the sigmoid activation function. NeuroImage. 2008;42(1):147–157. 10.1016/j.neuroimage.2008.04.239 18547818

[75] Deco G, Jirsa VK, Robinson PA, Breakspear M, Friston K. The Dynamic Brain: From Spiking Neurons to Neural Masses and Cortical Fields. PLOS Computational Biology. 2008;4(8):e1000092 10.1371/journal.pcbi.1000092 18769680 PMC2519166

[76] Breakspear M, Terry JR, Friston KJ. Modulation of excitatory synaptic coupling facilitates synchronization and complex dynamics in a biophysical model of neuronal dynamics. Network: Computation in Neural Systems. 2003;14(4):703–732. 10.1088/0954-898X_14_4_305 14653499

[77] Langdon AJ, Breakspear M, Coombes S. Phase-locked cluster oscillations in periodically forced integrate-and-fire-or-burst neuronal populations. Physical Review E. 2012;86(6):061903 10.1103/PhysRevE.86.061903 23367972

[78] Coombes S. Dynamics of synaptically coupled integrate-and-fire-or-burst neurons. Physical Review E. 2003;67(4):041910 10.1103/PhysRevE.67.041910 12786399

[79] Shine JM, van den Brink RL, Hernaus D, Nieuwenhuis S, Poldrack RA. Catecholaminergic manipulation alters dynamic network topology across cognitive states. Network Neuroscience. 2018;2(3):381–396. 10.1162/netn_a_00042 30294705 PMC6145851

[80] Smith SM, Miller KL, Salimi-Khorshidi G, Webster M, Beckmann CF, Nichols TE, et al Network modelling methods for FMRI. NeuroImage. 2011;54(2):875–891. 10.1016/j.neuroimage.2010.08.063 20817103

[81] Ramsey JD, Hanson SJ, Hanson C, Halchenko YO, Poldrack RA, Glymour C. Six problems for causal inference from fMRI. NeuroImage. 2010;49(2):1545–1558. 10.1016/j.neuroimage.2009.08.065 19747552

[82] Wu GR, Liao W, Stramaglia S, Ding JR, Chen H, Marinazzo D. A blind deconvolution approach to recover effective connectivity brain networks from resting state fMRI data. Medical Image Analysis. 2013;17(3):365–374. 10.1016/j.media.2013.01.003 23422254

[83] Rangaprakash D, Wu GR, Marinazzo D, Hu X, Deshpande G. Hemodynamic response function (HRF) variability confounds resting-state fMRI functional connectivity. Magnetic Resonance in Medicine. 2018;80(4):1697–1713. 10.1002/mrm.27146 29656446

[84] Stefanescu RA, Jirsa VK. A Low Dimensional Description of Globally Coupled Heterogeneous Neural Networks of Excitatory and Inhibitory Neurons. PLOS Computational Biology. 2008;4(11):e1000219 10.1371/journal.pcbi.1000219 19008942 PMC2574034

[85] Cohen JR, D’Esposito M. The Segregation and Integration of Distinct Brain Networks and Their Relationship to Cognition. The Journal of Neuroscience. 2016;36(48):12083–12094. 10.1523/JNEUROSCI.2965-15.2016 27903719 PMC5148214

[86] Shine JM, Breakspear M, Bell PT, Ehgoetz Martens KA, Shine R, Koyejo O, et al Human cognition involves the dynamic integration of neural activity and neuromodulatory systems. Nature Neuroscience. 2019;22(2):289–296. 10.1038/s41593-018-0312-0 30664771

[87] FitzHugh R. Impulses and physiological states in theoretical models of nerve membrane. Biophysical Journal. 1961;1(6):445–466. 10.1016/s0006-3495(61)86902-6 19431309 PMC1366333

[88] Kotter R. Online Retrieval, Processing, and Visualization of Primate Connectivity Data From the CoCoMac Database. Neuroinformatics. 2004;2(2):127–44. 10.1385/NI:2:2:127 15319511

[89] Rüemelin W. Numerical treatment of stochastic differential equations. SIAM Journal on Numerical Analysis. 1982;19(3):604–613. 10.1137/0719041

[90] Shine JM. Gain_topology; 2018. https://github.com/macshine/gain_topology.

[91] Lizier JT. JIDT: An Information-Theoretic Toolkit for Studying the Dynamics of Complex Systems. Frontiers in Robotics and AI. 2014;1:11 10.3389/frobt.2014.00011

[92] Takens F. Detecting strange attractors in turbulence In: Rand D, Young LS, editors. Dynamical Systems and Turbulence, Warwick 1980. vol. 898 of Lecture Notes in Mathematics. Berlin / Heidelberg: Springer; 1981 p. 366–381. Available from: 10.1007/bfb0091924.

[93] Faes L, Nollo G, Porta A. Information-based detection of nonlinear Granger causality in multivariate processes via a nonuniform embedding technique. Physical Review E. 2011;83:051112 10.1103/PhysRevE.83.051112 21728495

[94] Garland J, James RG, Bradley E. Leveraging information storage to select forecast-optimal parameters for delay-coordinate reconstructions. Physical Review E. 2016;93:022221 10.1103/PhysRevE.93.022221 26986345

[95] Erten EY, Lizier JT, Piraveenan M, Prokopenko M. Criticality and Information Dynamics in Epidemiological Models. Entropy. 2017;19(5):194 10.3390/e19050194

[96] Brodski-Guerniero A, Naumer MJ, Moliadze V, Chan J, Althen H, Ferreira-Santos F, et al Predictable information in neural signals during resting state is reduced in autism spectrum disorder. Human Brain Mapping. 2018;39(8):3227–3240. 10.1002/hbm.24072 29617056 PMC6866422

[97] Bossomaier T, Barnett L, Harré M, Lizier JT. An Introduction to Transfer Entropy: Information Flow in Complex Systems. Cham, Switzerland: Springer International Publishing; 2016. Available from: 10.1007/978-3-319-43222-9.

[98] Vicente R, Wibral M, Lindner M, Pipa G. Transfer Entropy–a Model-free Measure of Effective Connectivity for the Neurosciences. Journal of Computational Neuroscience. 2011;30(1):45–67. 10.1007/s10827-010-0262-3 20706781 PMC3040354

[99] Wibral M, Rahm B, Rieder M, Lindner M, Vicente R, Kaiser J. Transfer entropy in magnetoencephalographic data: quantifying information flow in cortical and cerebellar networks. Progress in Biophysics and Molecular Biology. 2011;105(1-2):80–97. 10.1016/j.pbiomolbio.2010.11.006 21115029

[100] Lizier JT, Heinzle J, Horstmann A, Haynes JD, Prokopenko M. Multivariate information-theoretic measures reveal directed information structure and task relevant changes in fMRI connectivity. Journal of Computational Neuroscience. 2011;30(1):85–107. 10.1007/s10827-010-0271-2 20799057

[101] Ito S, Hansen ME, Heiland R, Lumsdaine A, Litke AM, Beggs JM. Extending Transfer Entropy Improves Identification of Effective Connectivity in a Spiking Cortical Network Model. PLoS ONE. 2011;6(11):e27431 10.1371/journal.pone.0027431 22102894 PMC3216957

[102] Stramaglia S, Wu GR, Pellicoro M, Marinazzo D. Expanding the transfer entropy to identify information subgraphs in complex systems. In: 2012 Annual International Conference of the IEEE Engineering in Medicine and Biology Society. IEEE; 2012. p. 3668–3671. 10.1109/EMBC.2012.6346762 23366723

[103] Nigam S, Shimono M, Ito S, Yeh FC, Timme N, Myroshnychenko M, et al Rich-Club Organization in Effective Connectivity among Cortical Neurons. Journal of Neuroscience. 2016;36(3):670–684. 10.1523/JNEUROSCI.2177-15.2016 26791200 PMC4719009

[104] Barnett L, Barrett AB, Seth AK. Granger causality and transfer entropy are equivalent for Gaussian variables. Physical Review Letters. 2009;103(23):238701 10.1103/PhysRevLett.103.238701 20366183

[105] Barnett L, Seth AK. Detectability of Granger causality for subsampled continuous-time neurophysiological processes. Journal of Neuroscience Methods. 2017;275:93–121. 10.1016/j.jneumeth.2016.10.016 27826091

[106] Vakorin VA, Krakovska OA, McIntosh AR. Confounding effects of indirect connections on causality estimation. Journal of Neuroscience Methods. 2009;184(1):152–160. 10.1016/j.jneumeth.2009.07.014 19628006

[107] Williams PL, Beer RD. Generalized Measures of Information Transfer. arXiv preprint arXiv:11021507. 2011;.

[108] Williams P, Beer R. Decomposing multivariate information. arXiv preprint arXiv:10042515. 2010;.

[109] Lizier JT, Bertschinger N, Jost J, Wibral M. Information Decomposition of Target Effects from Multi-Source Interactions: Perspectives on Previous, Current and Future Work. Entropy. 2018;20(4):307 10.3390/e20040307 PMC7512824 33265398

[110] Finn C, Lizier JT. Pointwise partial information decomposition using the specificity and ambiguity lattices. Entropy. 2018;20(4):297 10.3390/e20040297 PMC7512814 33265388

[111] Atay FM, Karabacak O. Stability of Coupled Map Networks with Delays. SIAM Journal on Applied Dynamical Systems. 2006;5(3):508–527. 10.1137/060652531

